# Phytoestrogens Enhance the Vascular Actions of the Endocannabinoid Anandamide in Mesenteric Beds of Female Rats

**DOI:** 10.1155/2012/647856

**Published:** 2012-01-29

**Authors:** Roxana N. Peroni, Tamara Abramoff, Isabel Neuman, Ernesto J. Podestá, Edda Adler-Graschinsky

**Affiliations:** ^1^Instituto de Investigaciones Farmacológicas, Consejo Nacional de Investigaciones Científicas y Técnicas, Universidad de Buenos Aires, Junín 956, 5° Piso, C1113AAD Buenos Aires, Argentina; ^2^Departamento de Bioquímica IIMHNO, Facultad de Medicina, Universidad de Buenos Aires, Paraguay 2155, 5° Piso C1121ABG Buenos Aires, Argentina

## Abstract

In rat isolated mesenteric beds that were contracted with NA as an in vitro model of the vascular adrenergic hyperactivity that usually precedes the onset of primary hypertension, the oral administration (3 daily doses) of either 10 mg/kg genistein or 20 mg/kg daidzein potentiated the anandamide-induced reduction of contractility to NA in female but not in male rats. Oral treatment with phytoestrogens also restored the vascular effects of anandamide as well as the mesenteric content of calcitonin gene-related peptide (CGRP) that were reduced after ovariectomy. The enhancement of anandamide effects caused by phytoestrogens was prevented by the concomitant administration of the estrogen receptor antagonist fulvestrant (2.5 mg/kg, s.c., 3 daily doses). It is concluded that, in the vasculature of female rats, phytoestrogens produced an estrogen-receptor-dependent enhancement of the anandamide-vascular actions that involves the modulation of CGRP levels and appears to be relevant whenever an adrenergic hyperactivity occurs.

## 1. Introduction

Endocannabinoids contribute to reduce vascular contractility under pathological conditions where vascular responsiveness is altered. Hence, compounds that selectively modulate the action as well as the levels of endocannabinoids represent templates for potential new therapeutic strategies [1]. In this sense, the exogenous administration of the endocannabinoid anandamide is known to induce the decrease of blood pressure in spontaneously hypertensive rats [2] as well as in Wistar rats fed with a high-salt diet [3]. Inhibition of the fatty acid amide hydrolase, enzyme involved in intracellular anandamide degradation, normalizes the cardiovascular function in hypertensive rats without producing adverse metabolic effects [4]. Estrogens are also positive modulators of the anandamide effects at the vascular wall since they stimulate the release of anandamide from human endothelial cells [5] as well as potentiate the anandamide-induced vasorelaxations by increasing the bioavailability of the calcitonin-related peptide (CGRP) in the rat mesenteric vasculature [6]. This potent vasodilator peptide is released, at least in part, as a consequence of the activation of the transient receptor potential vanilloid type 1 (TRPV1) by anandamide [7].

Phytoestrogens, such as genistein and daidzein, are able to activate estrogen receptors which confer to them weak estrogen-like activity [8]. They are associated with a favorable cardiovascular risk profile [9] and therefore constitute an interesting food-based alternative to the hormone replacement therapy during the menopausal transition in women [10]. Since in a variety of rat cell lines phytoestrogens inhibit anandamide uptake by blocking the fatty acid amide hydrolase [11], it is possible that phytoestrogens also regulate the vascular effects of anandamide. Hence, the aim of the present study was to elucidate whether oral administration of the soy-derived phytoestrogens genistein and daidzein modulates the anandamide-induced reductions of the contractions to NA in the rat isolated mesenteric bed that was used as an in vitro model of the adrenergic hyperactivity that usually precedes the onset of primary hypertension [12].

The hypothesis is that the endocannabinoid system could be another target, in addition to nitric oxide, prostanoids, and antioxidant defense genes, for the beneficial cardiovascular actions proposed for phytoestrogens [13, 14].

## 2. Materials and Methods

### 2.1. Animals

Male and female Sprague-Dawley rats were housed under a 12 : 12 h light: dark cycle, at controlled room temperature with food and water *ad libitum. *Experiments were conducted in accordance to the Guide for the Care and Use of Laboratory Animals of the National Research Council (USA, 1996). Adult female rats (8–10 weeks, 165–200 g body weight) were either bilaterally ovariectomized (OVX) or sham-operated through dorsal incision, under anaesthesia (40–60 mg/kg ketamine hydrochloride + 10 mg/kg xylazine hydrochloride). After 21 days of endogenous hormonal decline, the animals were randomly allocated to either drug-treated or vehicle-treated groups.

### 2.2. Animal Treatments

Dose and duration of treatment with phytoestrogens were selected on the basis that they reverted partially, but significantly the uterine atrophy caused by ovariectomy that is considered a parameter of estrogenic activity [15]. According to this, genistein (10 mg/kg) or daidzein (10–20 mg/kg) was administered by oral gavage (p.o.) once daily during three days. Drugs were dissolved in dimethylsulfoxide (residual concentration <1%) and were diluted in corn oil. Some intact as well as OVX female rats treated with phytoestrogen received concomitant subcutaneous administrations of the estrogen receptor antagonist 2.5 mg/kg fulvestrant (ICI 182,780) dissolved in ethanol and diluted in corn oil. All groups were analyzed by comparison with the corresponding vehicle-treated animals.

### 2.3. Mesenteric Vascular Bed Preparation

Adult male (250–350 g) and female (230–350 g) Sprague-Dawley rats were anaesthetized with urethane (1.2 g kg^−1^ body weight), the abdomen was opened, and the mesenteric vascular bed was cannulated and removed according to [16]. The isolated mesenteric bed was transferred to a perspex chamber and perfused with the Krebs solution at 37°C bubbled with 95% O_2_ plus 5% CO_2_ at a constant flow rate of 2 mL/min, maintained by a peristaltic pump. Changes in vascular resistance were measured as changes in perfusion pressure and recorded through a Statham pressure transducer connected to a Grass polygraph. Up to nine consecutive, 20 min apart bolus injections of noradrenaline (NA) were performed in one preparation because the short contractile responses induced by this drug are highly reproducible. On the contrary, the sustained contractions that are obtained whenever the agonists are added to the perfusate are difficult to reproduce in the same preparation [17]. 

Table 1 shows that the contractile responses to NA in the mesenteric bed had similar magnitudes between the groups (e.g., males and intact and ovariectomized females). Moreover, phytoestrogen treatment did not modify per se either the basal tone or the reactivity to NA; respect to the vehicle-treated groups.

To evaluate anandamide-induced effect, after the first NA bolus injection considered as control, cumulative anandamide concentrations were perfused during 20 min, and the responsiveness to NA (a submaximal pressor effect, i.e., 40 to 60 mm Hg) was challenged on every one concentration. Anandamide was dissolved in ethanol (<0.1%), and further dilutions were made in the Krebs solution. No effects on basal tone of mesenteries isolated from either male or female rats were observed for any concentration of anandamide.

### 2.4. Immunohistochemistry for CGRP

The experiments were performed according to [6]. Deeply anaesthetized rats were fixed by transcardiac perfusion with PBS containing 4% w/v paraformaldehyde, and mesenteric vascular beds were dissected. The endogenous peroxidase activity was blocked with 1% H_2_O_2_, and the preparations were permeabilized with 0.2% Triton X-100. Overnight incubation at room temperature with 1/3,000 anti-CGRP antibody (Sigma Aldrich, St. Louis, MI, USA) and 1 h incubation with 1/200 goat anti-rabbit peroxidase conjugated antibody (Sigma Aldrich, St. Louis, MI, USA) were performed. Several branches of each mesenteric bed were not incubated with the primary antibody to obtain the corresponding controls. Peroxidase activity was evidenced with diaminobenzidine in an *Eclipse *50*i* NIKON light microscope equipped with a video camera Nikon DS-SM. All groups were simultaneously processed to prevent interassay differences. CGRP-immunoreactive fibers were quantified in second arterial branches. Relative area (stained/total area) per field and the difference between anti-CGRP-incubated tissues and the corresponding controls were measured. Morphometric analysis was performed with Image J software (1.34 S National Institutes of Health USA).

### 2.5. Drugs

(−)-Noradrenaline bitartrate, genistein, and daidzein were obtained from Sigma-Aldrich (St Louis, MI, USA). Fulvestrant (Faslodex) was kindly donated by Astra Zeneca. Anandamide was purchased from Cayman Chemical (Ann Arbor, MI, USA).

### 2.6. Statistical Analysis

Data are presented as the mean  ± SEM (*n* = 4 to 6)  of the percent reductions of the initial contraction to NA and were analyzed by two-way analysis of variance followed by Bonferroni's *post hoc t-test*. One-way analysis of variance followed by Dunnett's multiple comparison tests were performed for the immunohistochemical assays as well as for noradrenaline-induced contractions. In all the cases, a *P* < 0.05  was considered as significant. 

## 3. Results

**Figure 1:**
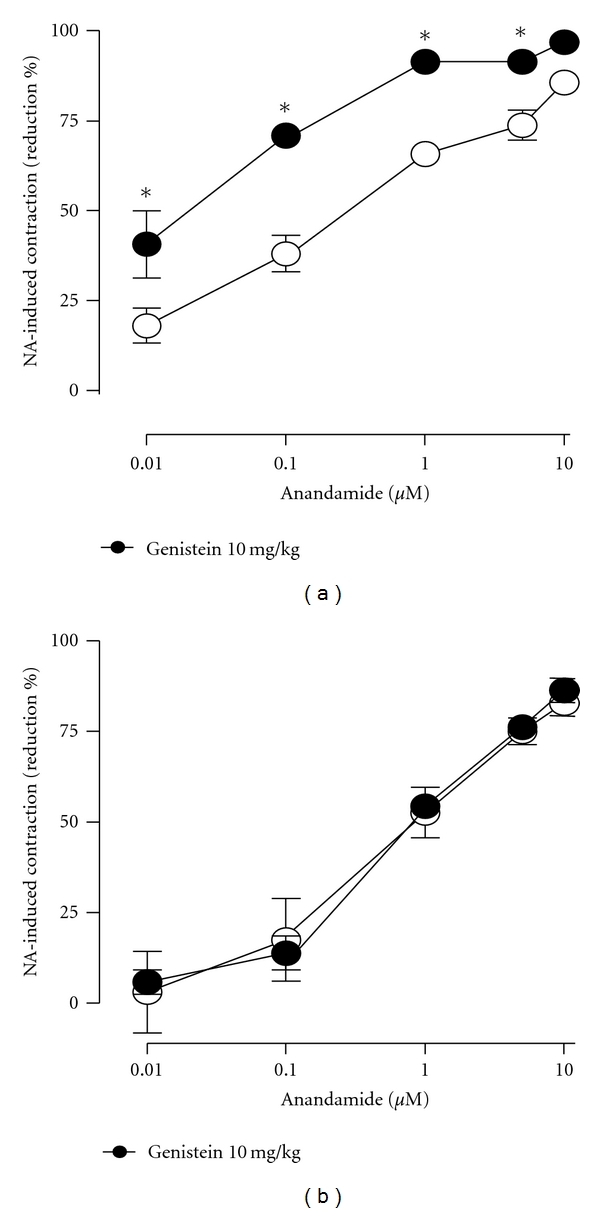
Effects of 3-day oral administration of 10 mg/kg genistein (filled circles) on anandamide-induced reductions of contractile responses to NA in mesenteric vascular beds isolated from female (a) as well as male (b) rats. Vehicles are depicted in open circles. *P < 0.05 when treatment with genistein was compared to the corresponding vehicle.

As shown in Figure 1, the endocannabinoid anandamide reduced, in a concentration-dependent manner, the transient contractions elicited by 10 nmol NA in mesenteric beds isolated from either male or female Sprague-Dawley rats. The oral administration of the phytoestrogen genistein (10 mg/kg; daily during 3 days) significantly potentiated the effect of anandamide in mesenteric beds isolated from female but not from male mesenteric beds. 

**Figure 2:**
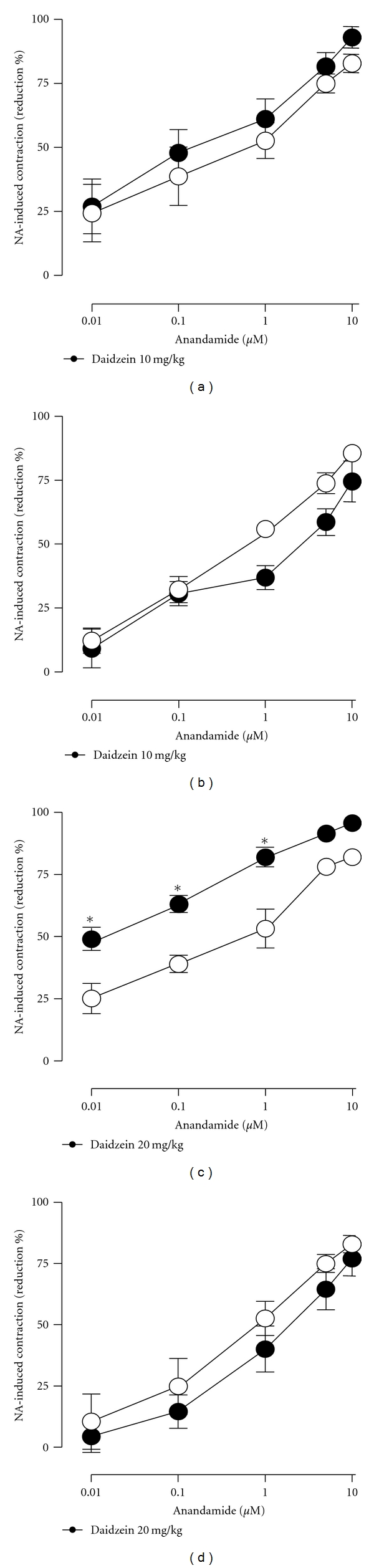
Effects of 3-day oral administration of either 10 mg/kg or 20 mg/kg daidzein (filled circles) on anandamide-induced reductions of contractile responses to NA in mesenteric vascular beds isolated from female (a) and (c) as well as male (b) and (d) rats. Vehicles are depicted in open circles. *P < 0.05  when treatment with daidzein was compared to the corresponding vehicle.

In turn, a 3-day treatment with the soy-derived phytoestrogen daidzein did not modify the anandamide-induced reduction of contractile responses when administered at a dose of 10 mg/kg to either female (Figure 2(a)) or male rats (Figure 2(b)) but did significantly increase the anandamide effects in mesenteries isolated from female rats when dose was scaled up to 20 mg/kg (Figure 2(c)).

The oral administration of the phytoestrogen genistein (10 mg/kg; daily during 3 days) significantly potentiated the effect of anandamide in mesenteric beds isolated from female but not from male mesenteric beds.

**Figure 3:**
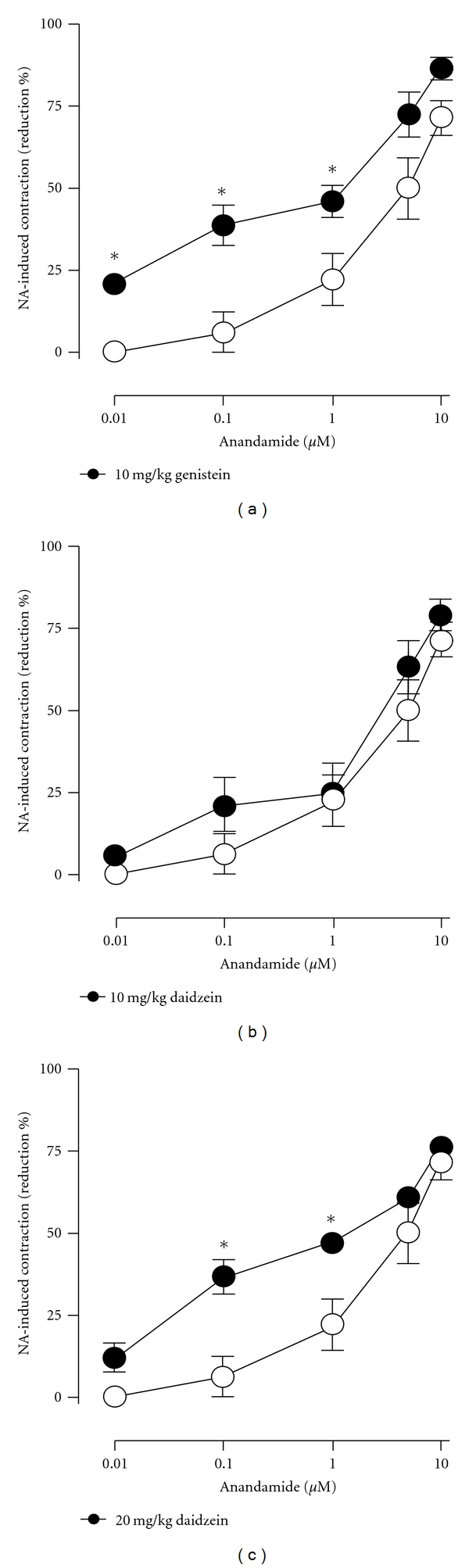
Effects of 3-day administration of 10 mg/kg genistein in (a), 10 mg/kg daidzein in (b), and 20 mg/kg daidzein in (c) on the anandamide-induced reductions of contractile responses to NA in mesenteric beds isolated from ovariectomized female rats. Vehicles are depicted in open circles. *P < 0.05 when treatments were compared to the corresponding vehicles.

Twenty-one days after ovariectomy, the ability of anandamide to decrease the contractile response to NA in mesenteric arteries declined significantly (compare intact females in Figure 1 and untreated OVX females in Figure 3; *P* < 0.001). Figure 3 shows that a 3-day treatment with either 10 mg/kg genistein or 20 mg/kg daidzein could restore anandamide-induced vascular effects in OVX rats. On the other hand, no effects were evidenced by treatment with 10 mg/kg daidzein (Figure 3(b)). 

**Figure 4:**
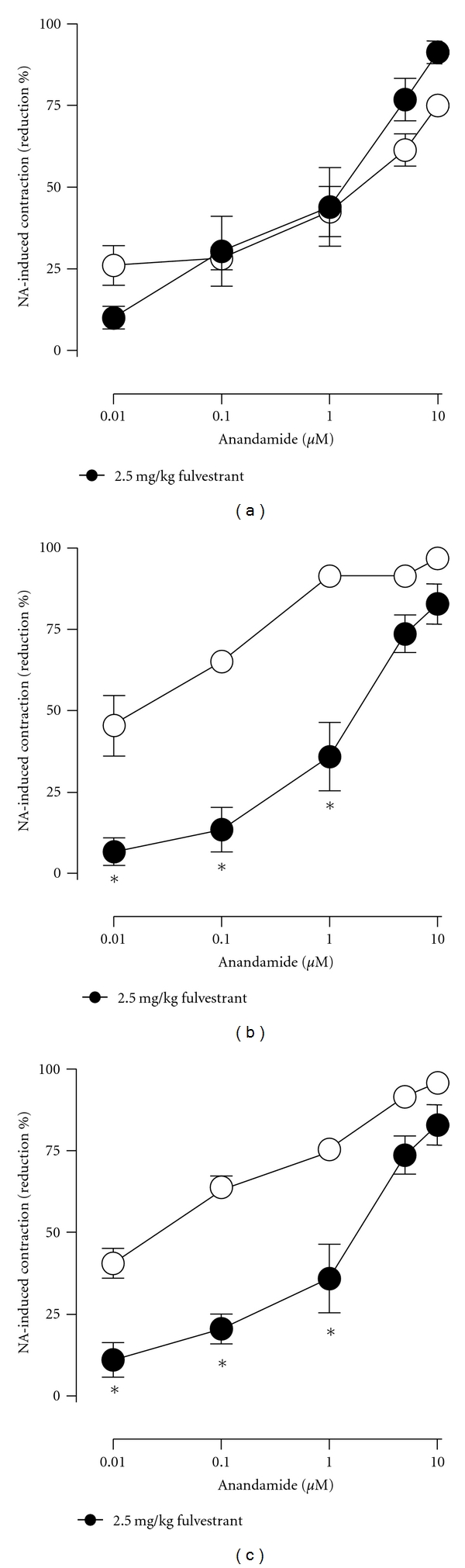
Effects of 3-day s.c. administration of 2.5 mg/kg of fulvestrant on the anandamide-induced reductions of contractile responses to NA in untreated (a), genisteintreated (10 mg/kg; (b)), or daidzein-treated (20 mg/kg; (c)) intact female rats. Either fulvestrant (filled circles) or its vehicle (open circles) were administered concomitantly with the corresponding phytoestrogen treatment. *P < 0.05 when phytoestrogen-treated were compared against phytoestrogen plus fulvestrant-treated intact female rats.

**Figure 5:**
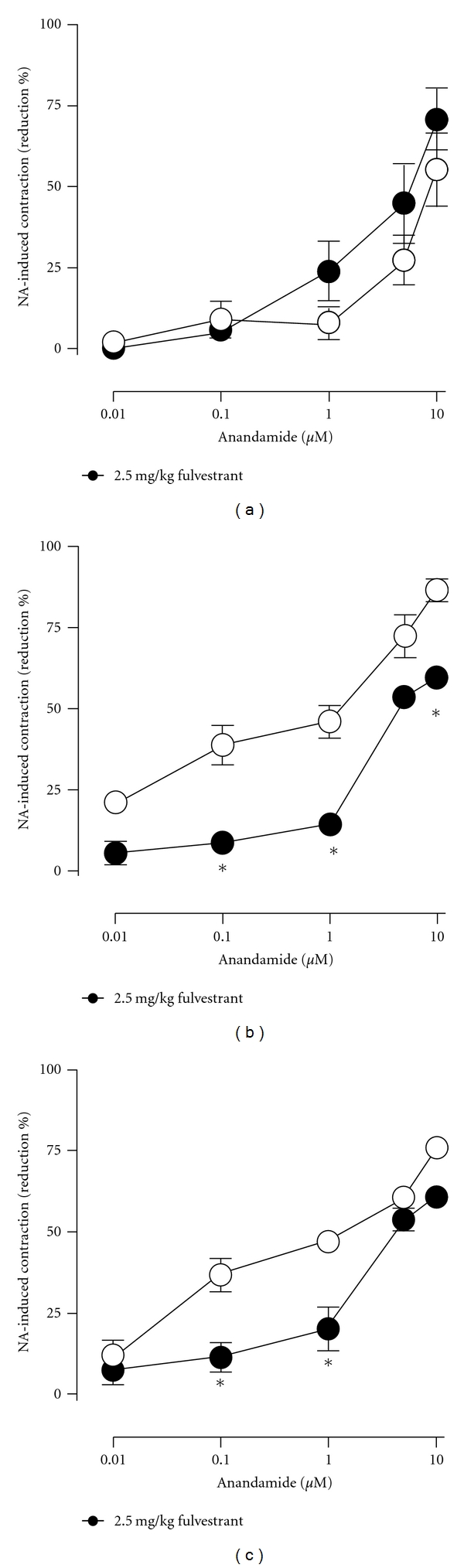
Effects of 3-day s.c. administration of 2.5 mg/kg of fulvestrant on the anandamide-induced reductions of contractile responses to NA in untreated (a), genistein-treated (10 mg/kg; (b)), or daidzein-treated (20 mg/kg; (c)) OVX female rats. Fulvestrant (filled circles) or its vehicle (open circles) were concomitantly administered with the corresponding phytoestrogen treatment. *P < 0.05  when phytoestrogen-treated were compared against phytoestrogen plus fulvestrant-treated OVX female rats.

**Figure 6:**
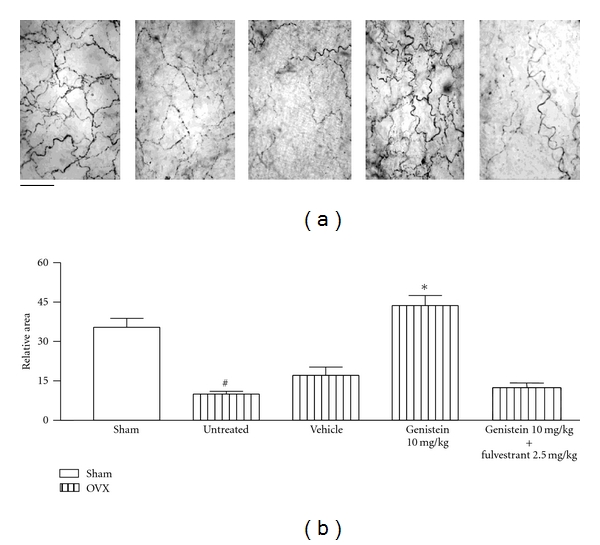
Immunohistochemistry for CGRP. (a) Representative microphotographs of CGRP-immunoreactive fibers surrounding mesenteric arteries in myenteric plexus preparations of sham-operated as well as of OVX female rats. Either genistein (10 mg/kg p.o.) or genistein plus fulvestrant (10 mg/kg p.o. and 2.5 mg/kg s.c., resp.) were administered daily during 3 days. The photomicrographs were captured at 400x magnification; the scale bar indicates 50 *μ*m. (b) Bars represent the mean ± SEM (*n* = 4) of relative morphometric units measured as stained area/total area. Specific immunoreactivity in every tissue was calculated as the difference between anti-CGRP-incubated and nonprimary antibody-incubated samples. ^#^ *P* < 0.001 between sham and untreated-ovariectomized female rats. *P < 0.01 between genistein-treated and either genistein plus fulvestrant-treated or the corresponding vehicle-treated OVX rats.

**Figure 7:**
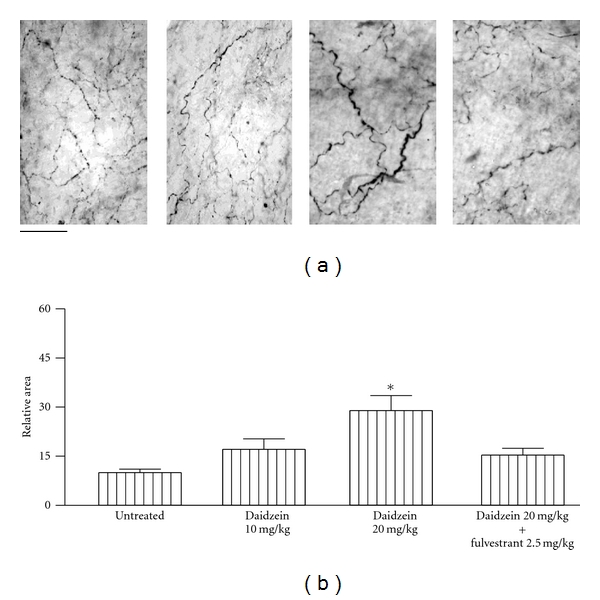
Immunohistochemistry for CGRP. (a) Representative microphotographs of CGRP-immunoreactive fibers surrounding mesenteric arteries in myenteric plexus preparations of OVX female rats. Daidzein (10 mg/kg or 20 mg/kg p.o.) or daidzein plus fulvestrant (20 mg/kg p.o. and 2.5 mg/kg s.c., resp.) were administered daily during 3 days. The photomicrographs were captured at 400x magnification; the scale bar indicates 50 *μ*m. (b) Bars represent the mean ± SEM (*n* = 4) of relative morphometric units measured as stained area/total area. Specific immunoreactivity in every tissue was calculated as the difference between anti-CGRP-incubated and nonprimary antibody-incubated samples. *P < 0.01 between daidzein-treated (20 mg/kg) and either daidzein plus fulvestrant-treated or untreated OVX rats.

The estrogen receptor antagonist fulvestrant (2.5 mg kg^−1^, s.c. during 3 days) did not modify *per se *the anandamide-induced reductions of precontracted arteries either in intact (Figure 4(a)) or in OVX (Figure 5(a)) females rats but significantly prevented the potentiation on anandamide-induced effects caused by either 10 mg/kg genistein or, 20 mg/kg daidzein in intact (Figures 4(b) and 4(c)) as well as in OVX female rats (Figures 5(b) and 5(c)). As shown in Figure 6, the density of the CGRP-containing fibers surrounding mesenteric arteries was markedly reduced by ovariectomy and significantly restored after a 3-day oral treatment with 10 mg/kg genistein. Moreover, when animals were concomitantly treated with genistein and fulvestrant, the enhancing effect of genistein was not observed. A similar profile was found after a 3-day oral treatment with 20 mg/kg but not with 10 mg/mg daidzein (Figure 7).

## 4. Discussion

The present study shows that oral administration of the soy-derived phytoestrogens genistein and daidzein daily during 3 days enhanced the decrease in the contractile responses to NA induced by anandamide in mesenteric arteries isolated from female but not from male rats. Taking into account that the anandamide effect is already greater in female mesenteries compared to males [16], the enhancing effect of the phytoestrogens shown here in female rats adds to the overall sex differentiation in this model. The tissue was selected because it represents a group of resistance vessels that greatly contributes to the maintenance of the total peripheral vascular resistance [17, 18]. Precontractions to NA were used to resemble the effects of the adrenergic hyperactivity in vascular tissues that usually precedes the onset of primary hypertension [12]. 

The observation that the anandamide-induced relaxations in the OVX rats were restored by a 3-day oral administration of phytoestrogens (present results) to the same extent as that produced by 17*β*-estradiol administration [16] suggests that a common site of action, namely, estrogen receptors (ER), could be involved in both cases. In support of this view is the finding that in intact as well as in OVX female rats the facilitatory effect of phytoestrogens was counteracted by the ER antagonist fulvestrant.

Moreover, a direct effect of phytoestrogens on the contractility to NA is precluded on the basis that no differences were observed in the responsiveness to NA between male and age-matched female rats after treatment with genistein or daidzein ([16] and present results). 

The fact that, as previously observed for 17*β*-estradiol [6], the 3-day oral treatment with either 10 mg/kg genistein or 20 mg/kg daidzein restored the decrease in the density of CGRP-containing perivascular fibers in mesenteries isolated from OVX rats could indicate that the modulation of CGRP levels contributes, among other factors, to the ability of phytoestrogens to potentiate the effect of anandamide in the mesenteric vasculature. In addition, this finding is supported on the basis that the presence of fulvestrant completely prevented the enhancing effect of phytoestrogens on CGRP perivascular levels. This hypothesis agrees with previous evidence showing that mesenteric availability of CGRP underlies the ability of anandamide to reduce the contractile responses to NA in mesenteric arteries [6]. Similarly, a cause-effect relationship between estrogen levels and CGRP arises from the observation that CGRP-containing fibers density is faded after ovariectomy and restored by estradiol treatment in sensory and perivascular neurons [6, 19]. Specifically related to phytoestrogens, it was reported that a diet with fujiflavone P40, a soybean isoflavone product, completely reverses the decrease in the levels of the mRNA coding for CGRP in dorsal root ganglion neurons, as well as the diminutions of the gastric tissue levels of CGRP in OVX rats [20]. In this sense, the lack of effect of oral treatment with 10 mg/kg daidzein agrees with the present observation that 10 mg/kg daidzein did not counteract the decrease in mesenteric CGRP content caused by OVX. This observation is consistent with previous evidence showing that genistein is 10 to 100 times more potent than daidzein and that this difference is linked to a higher affinity of genistein for ER [8], as observed for the modulation of the expression of enzymes that metabolize 17 *α*-estradiol in cultured MCF-7 cells [21].

On the other hand, the fact that phytoestrogens enhanced anandamide-induced effects selectively in the vasculature of female rats (present results) differs from the potentiation caused by 17*β*-estradiol in this tissue that is only observed in males [16]. This discrepancy could arise from the fact that the sex-related differences in the rat vasculature, which include variations in the density and distribution of ER subtypes [22], could only become evident when tissues are exposed to compounds, such as genistein and daidzein, that are known to possess a 1000-times lower estrogenic activity than estradiol and are supposed to act as ERbeta partial agonist [8]. In accordance to this, vasodilation to genistein but not to 17*β*-estradiol is enhanced in postmenopausal women suffering coronary heart disease that express high ERbeta in the vascular wall [23]. However, a more extensive analysis of the estrogen receptor subtypes involved in the vascular effects of anandamide, including variations in the density and distribution of ER subtypes in the mesenteric bed of male and female Sprague-Dawley rats, is necessary to reinforce this possibility.

Moreover, the lack of effect of phytoestrogens in males (present results) could rely on the fact that phytoestrogens modify male gonadal steroids levels [24, 25]. In support of the latter, it was reported that the control of anxiety-related behaviours produced by the systemic administration of phytoestrogens in male rats depends on the gonadal status [26]. 

At the molecular level, the fatty acid amidohydrolase (FAAH), the major anandamide-hydrolyzing enzyme, is a potential locus for an interaction between oestrogens and endocannabinoid signalling. The FAAH enzyme possesses an oestrogen response element in its genetic sequence, and translocation of the oestrogen receptor to the nucleus results in inhibition of FAAH transcription that leads to an increase in the anandamide signalling [27]. Since genistein and daidzein inhibit the fatty acid amidohydrolase in vitro [11], the possibility exists that, at least in part, the modulatory effect of phytoestrogens on the anandamide-induced reductions of the contractility to NA could involve the increase of anandamide levels. Nevertheless, this possibility is likely to be precluded since the FAAH inhibitor PMSF is devoid of effects on anandamide-induced vasodilations in the rat mesenteric vascular beds isolated from either sex [16], as well on anandamide actions in other rat tissues, namely, the brain [28].

In conclusion, this is the first evidence that the soy-derived phytoestrogens, genistein and daidzein, modulate positively the reduction of the contractility to NA produced by anandamide in the mesenteric vasculature and supports the hypothesis that the endocannabinoid system could be a target for the beneficial cardiovascular actions of dietary phytoestrogens, as proposed before for estrogens [5]. 

Finally, the present results give further support to the view that a dietary intervention with an isoflavone-enriched soy extract, acting at the cardiovascular level with minimal impact in the reproductive tract, could have implications for women's cardiovascular health, for example, enhancing the vasorelaxation of small arteries whenever increased adrenergic hyperactivity occurs [23].

## Figures and Tables

**Table 1:** Noradrenaline-induced contractions.

Group	*n*	NA-induced contraction (mm Hg)
*Male rats*		
Control	6	51.39 ± 8.46
Vehicle for phytoestrogens	7	52.50 ± 4.72
10 mg/kg genistein	4	43.75 ± 6.38
10 mg/kg daidzein	5	62.5 ± 9.68

*Intact female rats*		
Control	6	53.54 ± 8.08
Vehicle for phytoestrogens	5	47.68 ± 6.67
10 mg/kg genistein	4	51.88 ± 4.72
10 mg/kg daidzein	6	55.42 ± 5.02
20 mg/kg daidzein	5	53.80 ± 1.70

*Ovariectomized female rats*		
Control	8	52.81 ± 3.61
Vehicle for phytoestrogens	4	41.25 ± 6.49
Vehicle for 17*β*-oestradiol	6	43.76 ± 4.55
10 mg/kg genistein	4	56.25 ± 11.48
10 mg/kg daidzein	4	55.00 ± 8.42
20 mg/kg daidzein	4	47.50 ± 4.50
450 *μ*g/kg 17*β*-oestradiol	6	52.50 ± 9.64
